# Cross-Species Modeling Identifies Gene Signatures in Type 2 Diabetes Mouse Models Predictive of Inflammatory and Estrogen Signaling Pathways Associated with Alzheimer’s Disease Outcomes in Humans

**DOI:** 10.1142/9789819807024_0031

**Published:** 2025

**Authors:** Brendan K. Ball, Elizabeth A. Proctor, Douglas K. Brubaker

**Affiliations:** 1 Weldon School of Biomedical Engineering, Purdue University, West Lafayette, IN, USA.; 2 Department of Neurosurgery, Penn State College of Medicine, Hershey, PA, USA.; 3 Department of Pharmacology, Penn State College of Medicine, PA, USA.; 4 Department of Biomedical Engineering, Penn State University, State College, PA, USA.; 5 Center for Neuroengineering, Penn State University, State College, PA, USA.; 6 Department of Engineering Science & Mechanics, Penn State University, State College, PA, USA.; 7 Center for Global Health & Diseases, Department of Pathology, School of Medicine, Case Western Reserve University, Cleveland, OH, USA.; 8 Blood Heart Lung Immunology Research Center, University Hospitals, Cleveland, OH, USA.

**Keywords:** Alzheimer’s disease, type 2 diabetes, preclinical translation, cross-species modeling, systems biology

## Abstract

Alzheimer’s disease (AD), the predominant form of dementia, is influenced by several risk factors, including type 2 diabetes (T2D), a metabolic disorder characterized by the dysregulation of blood sugar levels. Despite mouse and human studies reporting this connection between T2D and AD, the mechanism by which T2D contributes to AD pathobiology is not well understood. A challenge in understanding mechanistic links between these conditions is that evidence between mouse and human experimental models must be synthesized, but translating between these systems is difficult due to evolutionary distance, physiological differences, and human heterogeneity. To address this, we employed a computational framework called translatable components regression (TransComp-R) to overcome discrepancies between pre-clinical and clinical studies using omics data. Here, we developed a novel extension of TransComp-R for multi-disease modeling to analyze transcriptomic data from brain samples of mouse models of AD, T2D, and simultaneous occurrence of both disease (ADxT2D) and postmortem human brain data to identify enriched pathways predictive of human AD status. Our TransComp-R model identified inflammatory and estrogen signaling pathways encoded by mouse principal components derived from models of T2D and ADxT2D, but not AD alone, predicted with human AD outcomes. The same mouse PCs predictive of human AD outcomes were able to capture sex-dependent differences in human AD biology, including significant effects unique to female patients, despite the TransComp-R being derived from data from only male mice. We demonstrated that our approach identifies biological pathways of interest at the intersection of the complex etiologies of AD and T2D which may guide future studies into pathogenesis and therapeutic development for patients with T2D-associated AD.

## 1. Introduction

Alzheimer’s disease (AD) is a progressive neurodegenerative disorder characterized by memory loss, confusion, and behavioral changes. With more than 6.9 million people living with AD in the United States^1^, $360 billion dollars in health and long-term care costs is expected to be spent in 2024, and projected to rise to $1 trillion by 2050^1^. As the prevalence of AD is expected to increase with the country’s aging population, developing effective therapeutics proven to treat or cure AD becomes urgent. Despite the rapid increase of AD cases, studies to develop therapeutics for AD is difficult^2,3^. This difficulty is in part due to the development of AD occurring decades before diagnosis^4^ and the multi-factorial nature of the disease^5–8^.

In efforts to identify risk factors for AD it was observed that individuals with type 2 diabetes (T2D), a metabolic condition distinguished by chronic hyperglycemia, have an elevated risk in developing AD^9,10^. The development of T2D occurs decades before the diagnosis of AD and is reported to increase the risk of dementia^11^. In the United States, more than 39 million people have T2D, and 116 million have pre-diabetes^12^. This population of people diagnosed with or at risk for developing T2D may face a heightened risk for developing AD in light of the comorbidity of the diseases^13,14^. In clinical studies, common features of both AD and T2D include chronic inflammation^15,16^, increased insulin resistance^17^, and alterations to mitochondria and energy metabolism^18,19^. Despite multiple studies supporting a link between T2D and AD risk, the biological mechanisms by which this occurs are not well understood.

A critical challenge in understanding the mechanistic links between these conditions is that evidence must be synthesized and translated between experiments in mouse models and human-based clinical studies. Translating information from pre-clinical models to human clinical contexts is difficult due to discrepancies in interspecies physiology^20^, timeline of disease development^21^, and heterogeneity of the human population^22^. In cases of precision medicine, where complex dependencies between clinical phenotypes are difficult to deconvolute, such as is the case with AD and T2D, there is an important role for computational approaches to resolve this heterogeneity into testable mechanistic hypotheses to guide therapeutic development^23–25^.

To overcome this challenge, we developed a computational framework termed translatable components regression (TransComp-R) to identify omics-based signatures in mouse models predictive of AD conditions in human^26–28^. The TransComp-R model works by projecting human omics data into a mouse principal component analysis (PCA) space, followed by linear regression of mouse principal components (PCs) against human disease outcomes to identify translatable mouse PCs. The gene signatures encoded within mouse PCs that best separate conditions between human AD and control outcomes can be interpreted using biological pathway analyses such as gene set enrichment analysis (GSEA). These informed pathways can then be validated through literature and experimental studies.

Here, we aimed to perform a cross-species analysis using publicly available mouse and human transcriptomic data to determine biological pathways by which T2D contributes to AD. We developed a novel extension of TransComp-R that integrated PCs from multiple murine disease models: AD, T2D, and co-occurrence of both diseases (ADxT2D) in a single computational model to compare the predictive power of different murine models of disease and identify mouse-specific features predictive of human AD status. We also modified the existing TransComp-R method by incorporating human demographic variables such as sex and age variables into our model to inform the selection of translatable mouse PCs and better position the insights from the cross-species model to specific human patient subsets, an important goal of precision medicine. Our method synthesizes mouse models with multiple disease etiologies with human information to prioritize biological pathways affected in disease and prospectively evaluate therapeutic avenues from pre-clinical to clinical contexts with high-throughput omics data.

## 2. Results

### 2.1. Selected mouse and human transcriptomic data were pre-processed for TransComp-R

Publicly available mouse (GSE152539)^29^ and human (GSE48350)^30,31^ datasets of microarrayed brain tissue samples were selected from Gene Expression Omnibus (GEO). The mouse dataset uniquely included conditions of solely AD, only T2D, and simultaneous occurrence of both diseases from the hippocampus. The mouse models consisted of six-month-old male *App*^*NL-F/NL-F*^ knock-ins responsible for heightened amyloid-beta in the brain (Swedish KM670/671NL, Iberian I716F) and wild type (C57BL/6J) mice were fed with either a high-fat diet (custom diet, 40% kcal from fat, and 0.15% from cholesterol) or regular diet (CA-1, 18.8% kcal from fat) for 12 months (n=3 per condition) for the respective disease groups. The human dataset contains demographic variables of sex and age along with the transcriptomic data of AD $\left(n=80\right)$ and control $\left(n=173\right)$ subjects from four brain regions: hippocampus, entorhinal cortex, superior frontal cortex, and post-central gyrus.

To prepare the data for the TransComp-R framework, both mouse and human transcriptomics datasets were matched for one-to-one homologs. From homolog matching, 13,428 genes were identified, and all other genes that did not have a matching homolog pair were excluded from the analysis. The human data was next filtered for the hippocampal region to account for brain-region variability. Any subjects below the age of 65 were removed from the study to reduce age bias (Table 1). Both datasets were individually log_2_ transformed and normalized by z-score per gene.

### 2.2. TransComp-R modeling separates human samples in mouse principal component space

**Figure 1.**
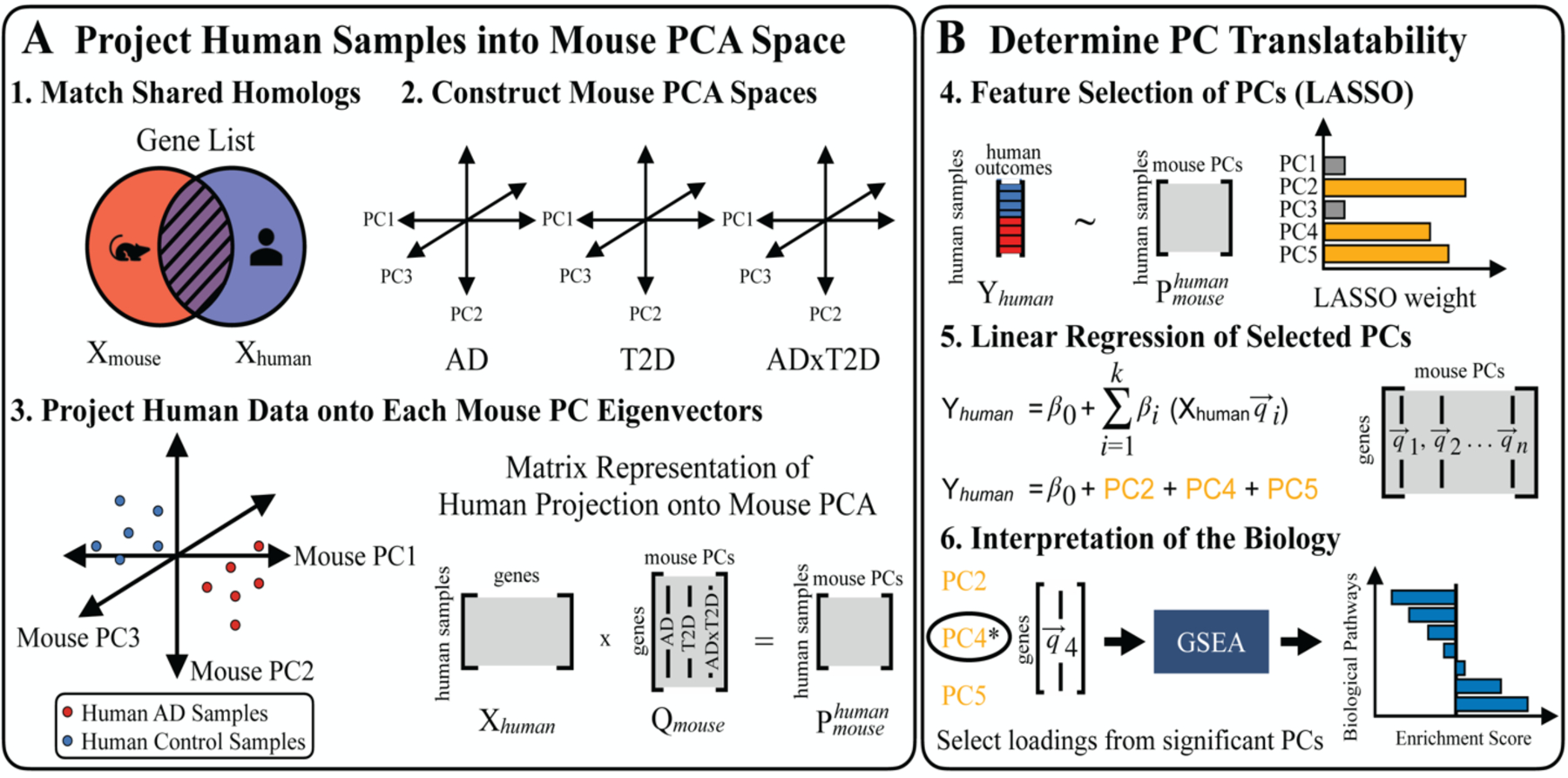
The TransComp-R computational approach. **(A)** Homolog gene pairs between human and mouse datasets are selected for analysis. Human samples are projected into mouse PCA spaces to combine mouse and human information. **(B)** Principal component translatability from mouse to human is determined by performing a GLM regression against human AD outcomes with PCs selected from LASSO. The loadings from the significant PCs are analyzed via GSEA to identify enriched biological pathways.

Here, we applied the TransComp-R methodology, with the incorporation of LASSO to select PCs most predictive of AD outcomes^26^. The TransComp-R model begins with the projection of human data into the mouse PCA space (Figure 1A), followed by the evaluation of mouse PC translatability through LASSO and generalized linear model (GLM) regression. The significant mouse PCs that can distinguish between human AD and control are interpreted by GSEA of the gene loading coefficients on each PC (Figure 1B). The biological pathways identified from GSEA can provide insight on human biology translated by mouse PCs, which can then be validated through follow-up experiments and literature review.

Implementing this approach, mouse data were separated into AD, T2D, and ADxT2D with controls prior to constructing separate PCA models, such that three groups of PCs encoded transcriptomic variation between healthy controls and AD, T2D, or ADxT2D mice. To avoid overfitting the mouse data, a threshold of 80% cumulative variance explained was set for each PCA, and as a result, a total of five PCs per disease group were selected (Supplementary Figure S1). Next, the human data was projected on the mouse PCA space.

We then trained four separate LASSO models to identify PCs most predictive of binarized human disease outcomes. Using the combined dataset containing rows of human and columns of T2D PCs, we incorporated progressively included human demographic variables associated with the respective human subjects in LASSO such that we examined: models of only mouse PCs, PCs with human sex, PCs with human age, and PCs with both human sex and age main effects. This approach allows us to include human demographic variables in a cross-species translation model, prioritizing not just mouse PCs, but also how mouse PCs capture the heterogeneity of human sex and age when predicting AD outcomes. The PCs were next selected based on 100 rounds of 5-fold cross-validation, where PCs with a significant LASSO coefficient in greater than half of the models were carried forward to the GLM.

**Figure 2.**
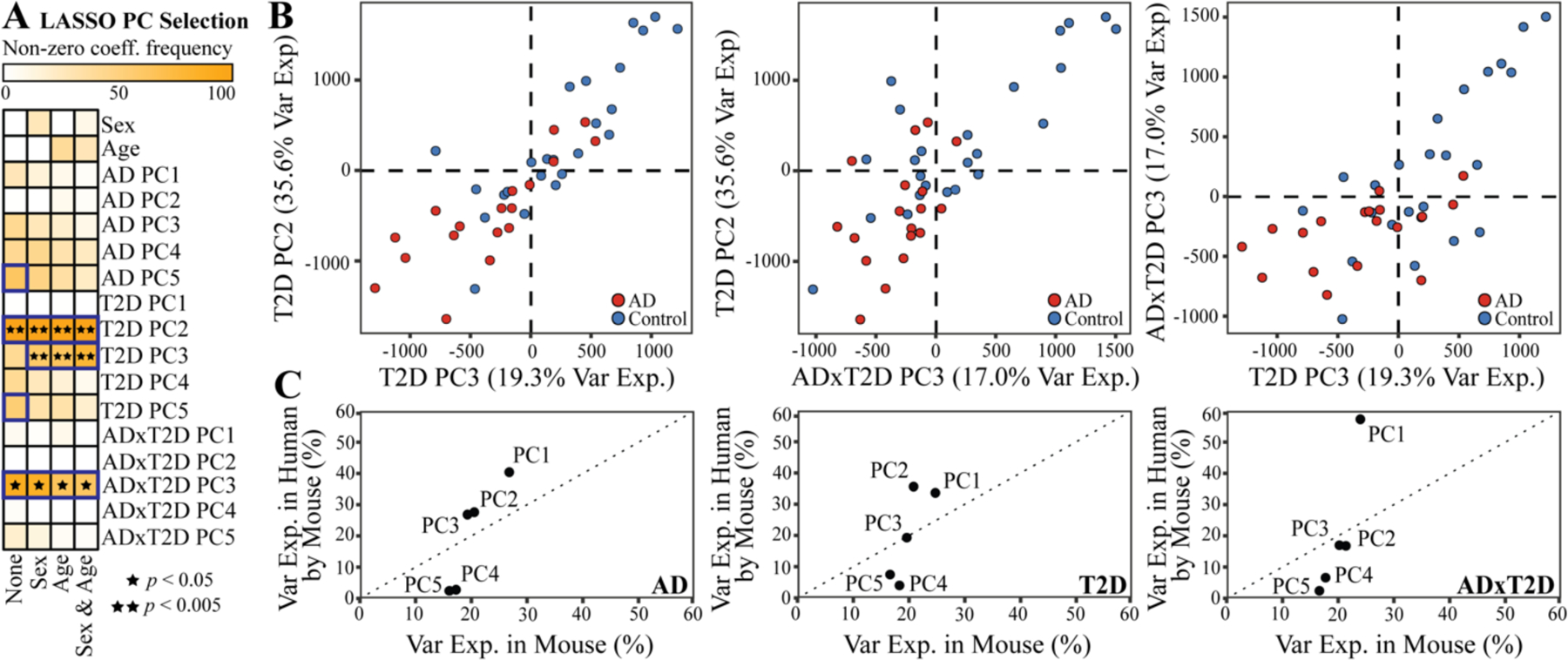
TransComp-R identifies translatable PCs predictive for AD outcomes in human. **(A)** Selection of PCs using LASSO across 100 rounds of 5-fold cross-validation. The four LASSO models included terms with just mouse PCs, PCs and human sex, PCs and human age, and PCs and human age and sex. PCs with a coefficient frequency greater than 50 rounds of 100 were selected for the GLM and regressed against binarized human disease outcomes (significance defined by simple regression model p value). **(B)** A principal component plot of human scores on the selected mouse T2D PC2, T2D PC3, and ADxT2D PC3 separating human control and AD outcomes **(C)** Mouse PCs were separated by disease cohort, comparing the variance explained in mice to the variance in humans explained by mouse respective mouse PCs.

From the LASSO models, we found T2D PC2 and ADxT2D PC3 to be consistently selected across all four LASSO models, while T2D PC3 was selected from all models except for the model with only mouse PC main effects (Figure 2A). Additionally, AD PC5 and T2D PC5 were selected from the LASSO model with only mouse PCs as main effect variables, but not in other LASSO models that included human demographic variables. The PCs identified by the LASSO model, which were encoded with transcriptomic variance, were next evaluated for their respective ability to discern between human AD and control status through GLM.

To evaluate the predictability of the selected mouse PCs for human AD, we constructed GLMs with all selected PCs predicting AD status in humans, but these multi-PC models were not significantly predictive due to multi-collinearity (Supplementary Figure S2). As a result, we constructed GLMs for each individual PC regressed against human disease outcomes. We found the three mouse PCs consistently selected from LASSO to be predictive of human AD outcomes individually (T2D PC2 p=0.0047, T2D PC3 p=0.0042, and ADxT2D PC3 p=0.0130) (Figure 2A). We also note that although AD PC5 and T2D PC5 satisfied the non-zero frequency greater than 50 in the LASSO model with only mouse PCs, the regression against human outcomes was not significant, and was excluded from further analysis (AD PC5 p=0.275, T2D PC5 p=0.443). Consistent LASSO selection of T2D PC2, T2D PC3, and ADxT2D PC3 as significant PCs indicates the importance of including human clinical and demographic variables in the TransComp-R model to detect translatable cross-species biology while controlling for clinical covariates.

We visualized the two T2D mouse PCs and one ADxT2D mouse PC that were identified by TransComp-R as predictive of human AD status (Figure 2B). In all three PCs, there was visible separation between the control and AD groups. We next compared the translatability of the selected mouse PCs to their ability to explain the variance in human data (Figure 2C). Comparing the proportion of PC variance explained in mouse to the variance explained in human by the same mouse PC, we found that T2D PC3 and ADxT2D PC3 explained a similar ratio, whereas mouse T2D PC2 explained almost double the variance in human by mouse than the mouse PCs alone. This could imply that certain pathways represented by mouse T2D PC3 and ADxT2D PC3 were conserved consistently across mice and humans, whereas mouse T2D PC2 may had a more pronounced effect in capturing information cross-species.

### 2.3. Mouse principal components selected genes contribute to human disease separation

**Figure 3.**
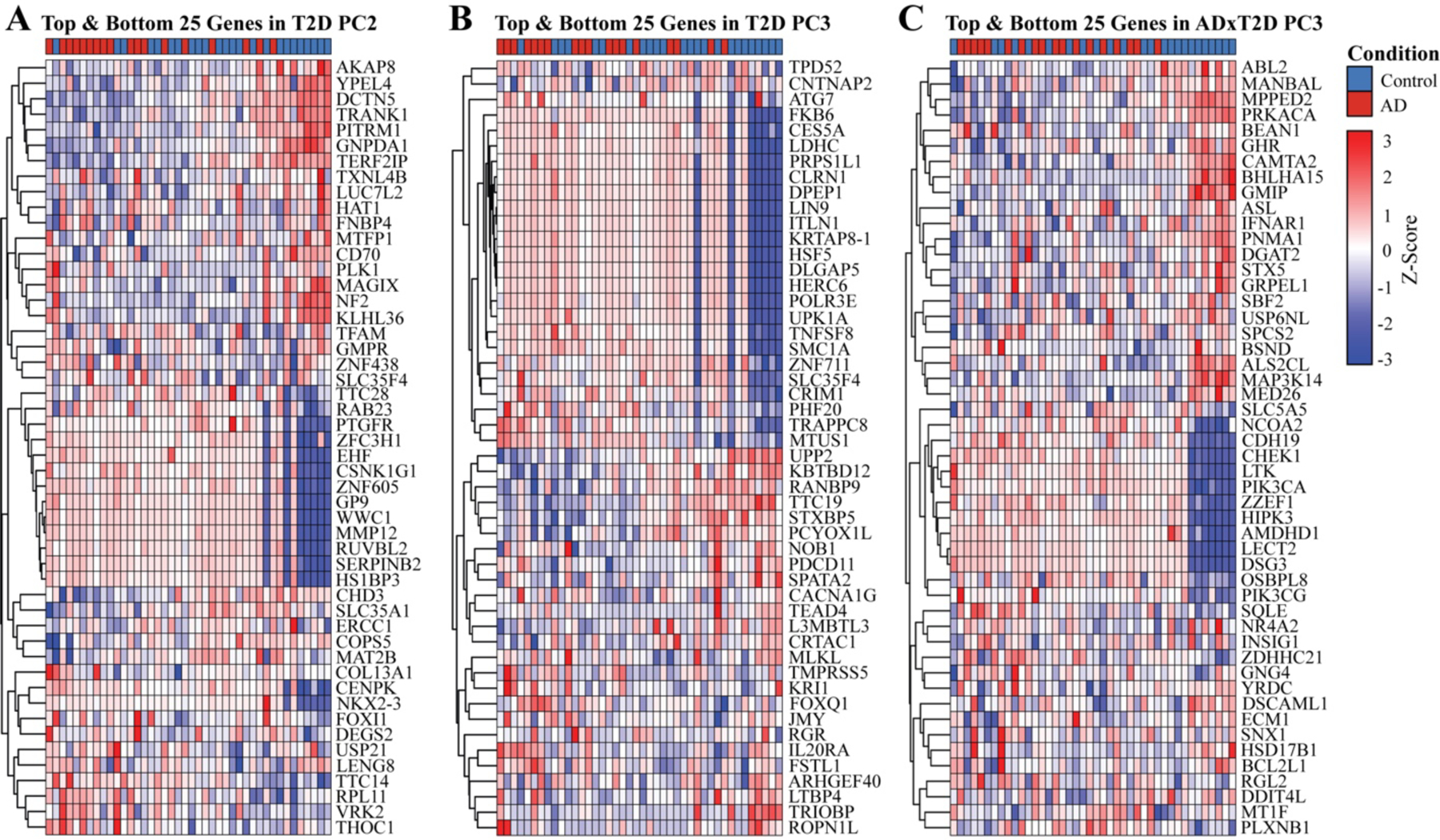
The top and bottom 25 genes of translatable PCs. Z-scored AD human transcriptomic data were filtered by genes with the 25 largest and smallest scores on **(A)** T2D PC2, **(B)** T2D PC3, and **(C)** ADxT2D PC3. Human samples were sorted by their respective PC scores with the most negative (left) to the most positive (right).

Having identified three mouse PCs predictive of human AD versus control status from TransComp-R, we were interested in isolating genes that were contributing to the separation between human AD and control subjects. Filtering for human genes ranked with the top and bottom 25 loadings within their respective PCs, we identified genes in the model predictive of AD and control in humans (Figure 3A–C). While no genes were shared across the top and bottom 25 ranked on the three mouse PCs, we observed distinct patterns of gene expression among human AD and control groups.

### 2.4. Gene set enrichment analysis identifies inflammatory and estrogen signaling pathways enriched in human Alzheimer’s disease outcome

**Figure 4.**
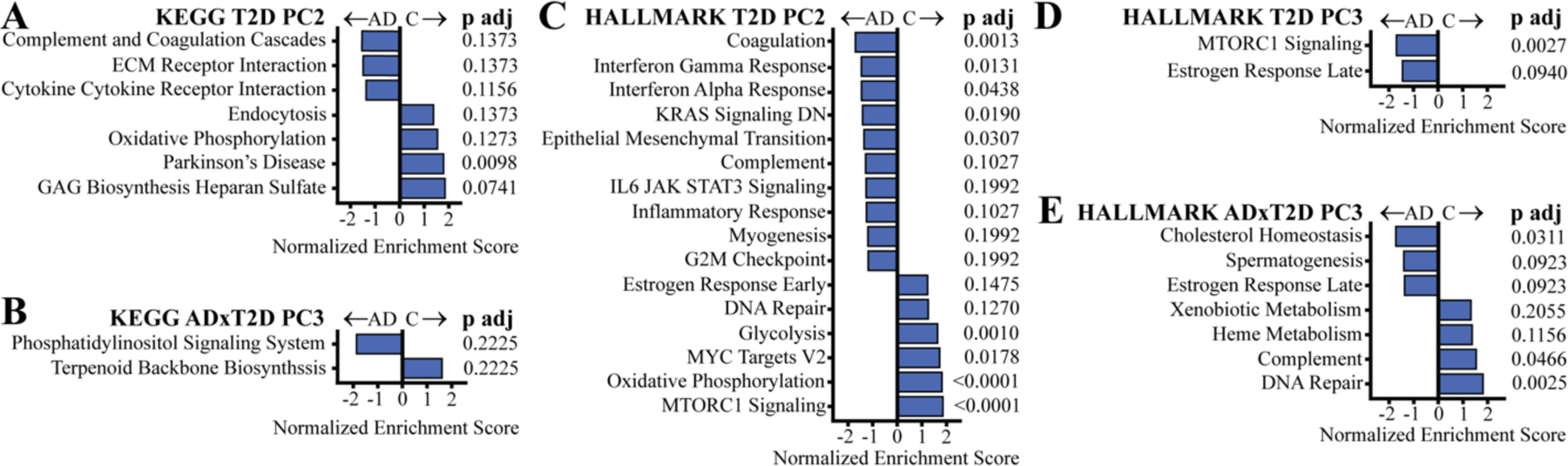
Enriched biological pathways identified from GSEA. Significant KEGG pathways from **(A)** T2D PC2 and **(B)** ADxT2D PC3. No significant pathways were enriched in T2D PC3. Significant Hallmark pathways were identified for **(C)** T2D PC2, **(D)** T2D PC3, and **(E)** ADxT2D PC3. Enriched pathways were defined by a Benjamini-Hochberg adjusted p value < 0.25. Pathways enriched for AD are displayed with a negative normalized enrichment score.

We performed GSEA on the selected T2D and ADxT2D PCs and identified pathways associated with inflammatory and estrogen signaling. From the KEGG database, we identified “Complement and Coagulation Cascades” and “Cytokine-Cytokine Receptor Interaction” on T2D PC2 (Figure 4A). On T2D PC3, the “Phosphatidylinositol Signaling System” was the only pathway found to be enriched for AD (Figure 4B). There were no significant KEGG pathways on T2D PC3. On the Hallmark database, we identified “Interferon Gamma Response,” “Interferon Alpha Response,” “IL6 JAK STAT Signaling,” and “Inflammatory Response” to be enriched for AD conditions by T2D PC2 (Figure 4C). Interestingly in the Hallmark database, we identified “Estrogen Response Early” was enriched for the control group in T2D PC2 (Figure 4C), while “Estrogen Response Late” was enriched for AD in T2D PC3 and ADxT2D PC3 (Figure 4D–E).

Based on our findings with the estrogen pathways, we were interested in distinguishing the genes that contributed to “Estrogen Response Early” and “Estrogen Response Late.” From GSEA, we identified 76, 77, and 49 core enrichment genes contributing to the estrogen-associated pathways in mouse T2D PC2, T2D PC3, and ADxT2D PC3, respectively. Comparing the genes that were contributing to the estrogen response, we found 23 shared genes between T2D PC3 and ADxT2D PC3 (*PDZK1*, *LLGL2*, *KLK11*, *TOP2A*, *PTGES*, *FARP1*, *NAB2*, *CISH*, *MEST*, *KIF20A*, *LTF*, *ISG20*, *IMPA2*, *DUSP2*, *PLAC1*, *PRKAR2B*, *TNNC1*, *OPN3*, *AREG*, *ATP2B4*, *AGR2*, *CALCR*, and *RABEP1*), 2 genes between T2D PC2 and ADxT2D PC3 (*DHCR7* and *MAPT*), 10 genes between T2D PC2 and T2D PC3 (*TPBG*, *FKBP4*, *GLA*, *NXT1*, *CD44*, *PGR*, *RAB31*, *AFF1*, *TFAP2C*, and *TJP3*), and 5 genes shared across all three mouse PCs (*SULT2B1*, *OVOL2*, *SIAH2*, *FDFT1*, and *RBBP8*) (Supplementary Figure S3). Additionally, 19 genes enriched in ADxT2D PC3, 39 genes enriched in T2D PC3, and 59 genes enriched in T2D PC2 did not overlap with any other mouse PCs.

### 2.5. Male mouse-derived principal components significantly stratify female Alzheimer’s disease and control groups in human subjects

**Figure 5.**
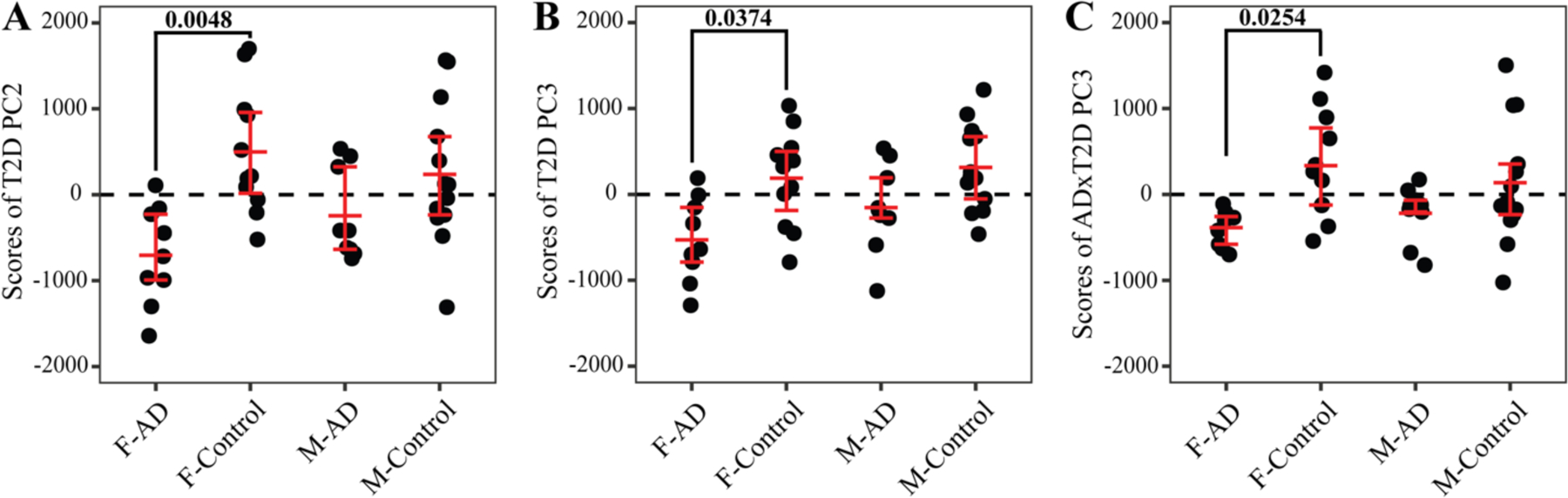
Comparison of sex and disease status among the translatable PCs. Scores of each PC were separated by female (F) AD, female control, male (M) AD, and male control for **(A)** T2D PC2, **(B)** T2D PC3, and **(C)** ADxT2D PC3. A Mann-Whitney pair-wise test corrected by the Benjamini-Hochberg method (FDR q value < 0.05) was used to determine the significance among the groups. The mean of the distribution is labeled with the interquartile range.

Expanding upon the potential sex-based predictability, we were curious to see if the model was able to distinguish sex and disease status by the PC scores. Here, we separated the scores of each mouse PC by human sex and AD status and found that mouse T2D PC2, T2D PC3, and ADxT2D PC3 significantly stratified human female AD and control groups, and not male AD and control groups, despite the mouse data originating from all male mice (Figure 5A–C). The ability of these PCs to distinguish between female AD and control groups shows the model’s ability in identifying human sex-based differences in the context of disease development. This is supported by the significance of the separation between the two groups (*p* value < 0.05).

## 3. Discussion

In this work, we aimed to uncover potential biological mechanisms that connected T2D as a risk factor for AD development using mouse and human transcriptomic data. An obstacle in understanding the links between these diseases, in which multifactorial mechanisms interact in humans and biological mechanisms are isolated in animal studies, is that information from mouse models and human-based studies must be synthesized to inform clinical and therapeutic decisions. Currently, translating information from pre-clinical models to patient-specific contexts is often difficult due to discrepancies in interspecies physiology^20^, timeline of disease development^21^, and heterogeneity of the human population^22^. To overcome these challenges, we innovated on TransComp-R to identify potential biological pathways from mouse PCs that are predictable for AD outcomes. In the TransComp-R workflow, we fused multiple mouse disease models in a single computational model together with human data containing demographic sex and age variables to predict outcomes in AD. With our computational model, we pinpointed potential biological pathways associated with AD, and identified sex-specific differences, despite the mouse disease models being representative of only males.

We identified inflammatory pathways that may link T2D as a risk factor for AD development. These links have the potential translational utility in bridging mouse and human biology to understand and develop therapeutic strategies for AD with T2D exacerbating factors. The mouse T2D PC2 identified several pathways on both KEGG and hallmark databases. From KEGG, “Complement and Coagulation Cascades” and “Cytokine-Cytokine Receptor Interaction” were enriched for AD. From the literature, studies report complement activation to be associated with insulin resistance and T2D^32–34^. Likewise, high complement levels are contributed by neurons and glial cells in AD^35,36^. In both T2D and AD, cytokines are found to actively participate in the progression of disease^37,38^.

Using complementary pathway databases, we identified “Interferon Gamma Response,” “Interferon Alpha Response,” “IL6 JAK STAT Signaling,” and “Inflammatory Response” pathways on mouse T2D PC2 enriched in human AD. Interferon gamma^39^ and alpha^40^, key cytokines in the innate immune response and response to viral infections, are altered in AD. However, we notice that interferon gamma^41^ is more associated with T2D, whereas interferon alpha^42,43^ is found to be elevated in subjects with type 1 diabetes instead^44^. IL6 JAK-STAT signaling has been reported to impair the insulin-degrading enzyme, a protein found to be associated with obesity and T2D^45^. In AD, IL6 signaling has been linked with cognitive impairment and metabolic alterations^46^. Collectively, these results may indicate that chronic inflammation could lead to downstream insulin resistance and cognitive deficits^47^.

Our results also indicate that estrogen signaling may serve as a potential connection between T2D and AD. From GSEA, ranked genes in ADxT2D PC3 and T2D PC3 both identified “Estrogen Response Late” as pathways enriched for AD, whereas “Estrogen Response Early” was enriched for human control by T2D PC2. Among the three PCs, 49 genes were enriched for ADxT2D PC3, 77 genes were enriched for T2D PC2, and 76 genes were enriched for T2D PC2. Of these, 59 were enriched in T2D PC2, and 23 were shared between ADxT2D PC3 and T2D PC3, in which we compared with previously published literature to potential associations with AD and T2D. Associated with AD in the mouse T2D PC2, we identified *MED13L*^48^ and *XBP1*^49^ connected to cognitive deficits, changes in mitochondrial metabolism (*PMAIP1*)^50^, inflammation (*RASGRP1*)^51^, and the expression of *NRIP1*^52^ reduced in AD. Similarly, we identified genes associated with insulin resistance (*FASN* and *FKBP5*)^53,54^, genetic variances of *RAPGEF1*^55^ and increased expression of *AQP3*^56^ related to T2D development. Interpreting genes shared across ADxT2D PC3 and T2D PC3, both PCs, we found *MEST*^57^ reported to alter Wnt signaling in AD, and *KIF20A*^58^, a gene found to be differentially expressed in AD. Likewise in T2D, we found *CISH*^59^ to be involved with gluconeogenesis, whereas beta-cells were preserved with upregulated *AGR2*^60^.

There were five genes shared across the three mouse PCs identifying estrogen signaling as a potential biological pathway, which included *SULT2B1*, *OVOL2*, *SIAH2*, *FDFT1*, and *RBBP8*. Of the five genes, all but *RBBP8* were reported to have connections to AD or T2D in literature. *SULT2B1*, part of the sulfotransferase family that catalyzes the sulfate conjunction of hormones and neurotransmitters, was found to be upregulated in AD rat models^61^. In a T2D study, *SULT2B1* overexpressed in the liver inhibited hepatic gluconeogenesis in two separate diabetic mouse models: one induced by high-fat diet, and another via leptin-deficiency (ob/ob)^62^. Other genes related to T2D include *OVOL2* and *SIAH2*. The presence of *OVOL2* was found to be linked with beta cell dedifferentiation, a mechanism linked with pancreatic dysfunction^63^, and *SIAH2* deficiency improved glucose and insulin tolerance^64^. Related to AD, inhibition of squalene synthase (*FDFT1*) inhibited by squalestatin reduced cellular prion protein in ScN2a, SMB, and ScGT1 (prion-infected cell lines)^65^, and protection against amyloid beta-induced synapse damage^66^. Further examination of these genes may be of potential interest to connect biological pathways between T2D and AD.

Interestingly in both diseases, previous studies report that estrogen may play a protective role in AD^67^ and T2D^68^. In AD, estrogen provides protection from amyloid-beta toxicity, a hallmark of AD pathology^69,70^. In females that experienced menopause, hormone therapy with estrogen has been found to reduce the risk of T2D onset^68^. Although studies indicate estrogen to be protective, others report that estrogen may be deleterious depending on the timing and onset of T2D^71,72^. These differences could be a result of the varying roles that different genes may have: some genes may contribute to disease when upregulated, while others may serve a protective role that can lead to disease if downregulated. This variability in genes could further explain the possible observation of estrogen appearing to have both harmful and protective effects. Therefore, further investigations are encouraged to further understand the role of estrogen as a shared pathway between AD and T2D.

Finally, we found that the mouse PCs defined by T2D (PC2 and PC3) and ADxT2D (PC3) were able to distinguish between female AD and control subjects. Despite the mouse groups being entirely male, our model detected sex-based differences in females. This is interesting because females are at a higher risk of developing AD than males^73^. Observing this result, as well as PCs showing enrichment for estrogen, may suggest that despite the widespread lack of female animals in preclinical research, our model is able to detect biological signals in male mice predictive of female human disease biology, thereby enhancing the retrospective utility of prior animal studies that fell short of equitable design. In the specific case of our models, the pathways we identified on the male mouse PC’s predictive of human female AD pathology implicate our model’s ability to translate transcriptomic signatures across human sex demographics.

There are limitations and opportunities to expand this study. Few research groups have explored the T2D-AD axis, and as a result, there are limited sample sizes available for mouse and human omics data. The incorporation of additional studies that satisfy the criteria of our selection process into the model may improve the confidence of these results. Second, our TransComp-R model only considers homologous gene pairs shared across mice and humans. As a result, we potentially omit genes in pre-processing that may be involved in the development of AD. Additionally, the GLM in our model only regresses against control or AD status without the incorporation of transient phases such as mild cognitive impairment. Finally, the TransComp-R framework has the opportunity to consider other clinical variables that may predict disease outcomes. Some additional factors include information on race, clinical neuropathological scores for AD severity, and current T2D biomarkers. Considering these potential factors may further enhance future cross-species modeling.

Our work expanded upon the existing TransComp-R framework to identify potential biological pathways in which T2D may exacerbate AD development. We show that mouse PCs from T2D and ADxT2D were most predictive of AD outcomes in human. Interestingly, mouse PC’s derived from mice with AD alone were not predictive of human AD, which may indicate that metabolic dysfunction encoded on the mouse T2D and T2DxAD PCs plays a more significant role in human AD biology than is typically accounted for. Indeed, these results encourage future applications of TransComp-R to overcome barriers of pre-clinical to human studies and identify affected biological pathways in AD or different diseases. The implications of this work for precision medicine can be expanded to other disease models that may be difficult to synthesize between pre-clinical experiments and clinical studies. This platform could synthesize various omics data from pre-clinical and patient-specific data to rationally select potential pathways to target, which may further enhance clinical studies or possible therapeutic avenues.

## 4. Materials and Methods

### 4.1. Data selection

Mouse and human datasets were selected with the criteria of matching hippocampal brain region, information containing AD and T2D conditions in the mouse dataset, human sample size greater than 12 per condition, and at least sex and age information in the human dataset. Additionally, datasets derived from similar sequencing platforms were prioritized. Search terms on GEO included phrases such as “hippocampus Alzheimer’s disease in human,” “mouse Alzheimer’s disease hippocampus,” and “mouse diabetes hippocampus.” Additional searches included the term “gene expression” on the GEO repository.

### 4.2. Pre-processing and normalization

Publicly available transcriptomic human and mouse data were obtained from the GEO repository using Bioconductor tools in R (*GEOquery* 2.70.0, *limma* 3.58.1, and *Biobase* 2.62.0)^74–76^. Before processing, all human subjects with a reported age below 65 years old were removed from the analysis to prevent bias from younger age groups. The imported datasets were log2 transformed, then human and mouse gene lists were matched for homologous pairs (*orthogene* 1.8.0)^77^. The two datasets were filtered for the hippocampal brain region. The genes were then internally normalized by z-score prior to TransComp-R modeling.

### 4.3. Cross-species modeling and variable selection

We applied TransComp-R by conducting PCA on the mouse data separated in AD, T2D, and ADxT2D groups with controls, such that three groups of PCs encoded transcriptomic variation between healthy controls and AD, T2D, or ADxT2D mice. To avoid overfitting, the number of PCs in its respective group was limited to an 80% cumulative variance explained. Human AD and control subjects were projected into mouse PCA space. Mouse PCs associated with AD outcomes in human were selected by performing LASSO across 100 rounds of 5-fold cross-validation regressing the human positions in mouse PC space against human disease status. Four sets of LASSO models were trained, including main effects of mouse PCs, PCs and human sex, PCs and human age, and PCs and human age and sex. PCs with a coefficient frequency greater than 50 of the 100 rounds were selected for GLMs with individual PCCs and human clinical covariates regressed against human AD outcomes. The significance of the PC was determined if the model p value was less than 0.05.

### 4.4. Variance explained in human by mouse principal components

Human data containing subject information and gene lists, as well as mouse PCs with a matching gene list, was used to calculate the variance explained by mouse in human. Using mouse PCs in the columns of Q, we projected the human data matrix X onto the PCs via matrix multiplication and calculated the percent variance of mouse in X explained by a given column $q_{i}$ of Q (with T representing the matrix transpose) as:

(1) $$\text{VarExpHuman}\left(q_{i}\right)=\frac{q_{i}^{T}\left[X^{T}X\right]q_{i}}{\sum \text{diag}\left(Q^{T}X^{T}XQ\right)}$$

### 4.5. Identifying genes contributing to human separation by mouse principal components

Genes contributing to the most positive and negative scores were identified by selecting loaded genes with the top 25 and bottom 25 scores in each of the selected PCs. The selected genes were then used to filter the gene list of the human dataset containing z-scored gene expression data. A heatmap, with the human subjects, clustered by their scores from the TransComp-R model, and the 50 total genes were visualized to compare gene expression between AD and control.

### 4.6. Gene set enrichment analysis

GSEA was performed on the loadings of selected PCs from the GLM in R (*msigdbr* 7.5.1, *fgsea* 1.28.0, and c*lusterProfiler* 4.10.1)^78–80^. From the Molecular Signatures Database, two human collections to perform GSEA included the KEGG and Hallmark databases. The parameters for the minimum gene set size and the maximum gene set size were set to 5 and 500, respectively. The tuning constant, epsilon, was established at 0. For both KEGG and Hallmarks databases, enriched biological pathways were determined significant if the Benjamini-Hochberg adjusted p value was less than 0.25.

### 4.7. Sex-based comparison across principal component scores

As an approach to compare predictability across sex, scores of selected PCs were separated by sex and disease categories. A Mann-Whitney pair-wise test was used to determine significance among four groups (AD females, control females, AD males, and control males). To correct for multiple comparisons, p values were adjusted with the Benjamini-Hochberg factor. An adjusted p value less than 0.05 was considered significant for the analysis.

## Supplementary Material

Supplementary Figures

Supplementary Material & Code Availability

All supplementary figures, documents, and code for this study are made publicly available at https://github.com/Brubaker-Lab/MouseHuman-TransCompR-T2D-AD.

## Figures and Tables

**Table 1.** Summary of the processed human data across disease condition, age, and sex.

Condition	Age (years)	Sex (%)		Total Sample
	(Mean ± SD)	Female	Male	Size $\left(n\right)$
Control	82.7 ± 9.5	11 (46%)	13 (54%)	24
Alzheimer’s Disease	84.3 ± 6.6	9 (50%)	9 (50%)	18
