# Supplementary material for: Cross-Species Modeling Identifies Gene Signatures in Type 2 Diabetes Mouse Models Predictive of Inflammatory and Estrogen Signaling Pathways Associated with Alzheimer’s Disease Outcomes in Humans
Source: Pac Symp Biocomput. Author manuscript; Available in PMC 2025 Dec 4. (PMC12674991; doi:10.1142/9789819807024_0031)
Supplement: Supplementary Figures [file NIHMS2115944-supplement-Supplementary_Figures.pdf]

Cross-Species Modeling Identifies Gene Signatures in Type 2 Diabetes Mouse Models Predictive of Inflammatory and Estrogen Signaling Pathways Associated with Alzheimer's Disease Outcomes in Humans

Authors and Affiliations: Brendan K. Ball[1], Elizabeth A. Proctor[2-6], Douglas K. Brubaker[7-8*]

1. Weldon School of Biomedical Engineering, Purdue University, West Lafayette, IN, USA
2. Department of Neurosurgery, Penn State College of Medicine, Hershey, PA, USA
3. Department of Pharmacology, Penn State College of Medicine, PA, USA
4. Department of Biomedical Engineering, Penn State University, State College, PA, USA
5. Center for Neuroengineering, Penn State University, State College, PA, USA
6. Department of Engineering Science & Mechanics, Penn State University, State College, PA, USA
7. Center for Global Health & Diseases, Department of Pathology, School of Medicine, Case Western Reserve University, Cleveland, OH, USA
8. Blood Heart Lung Immunology Research Center, University Hospitals, Cleveland, OH, USA.

***Corresponding Author:** dkb50@case.edu

Supplementary Information

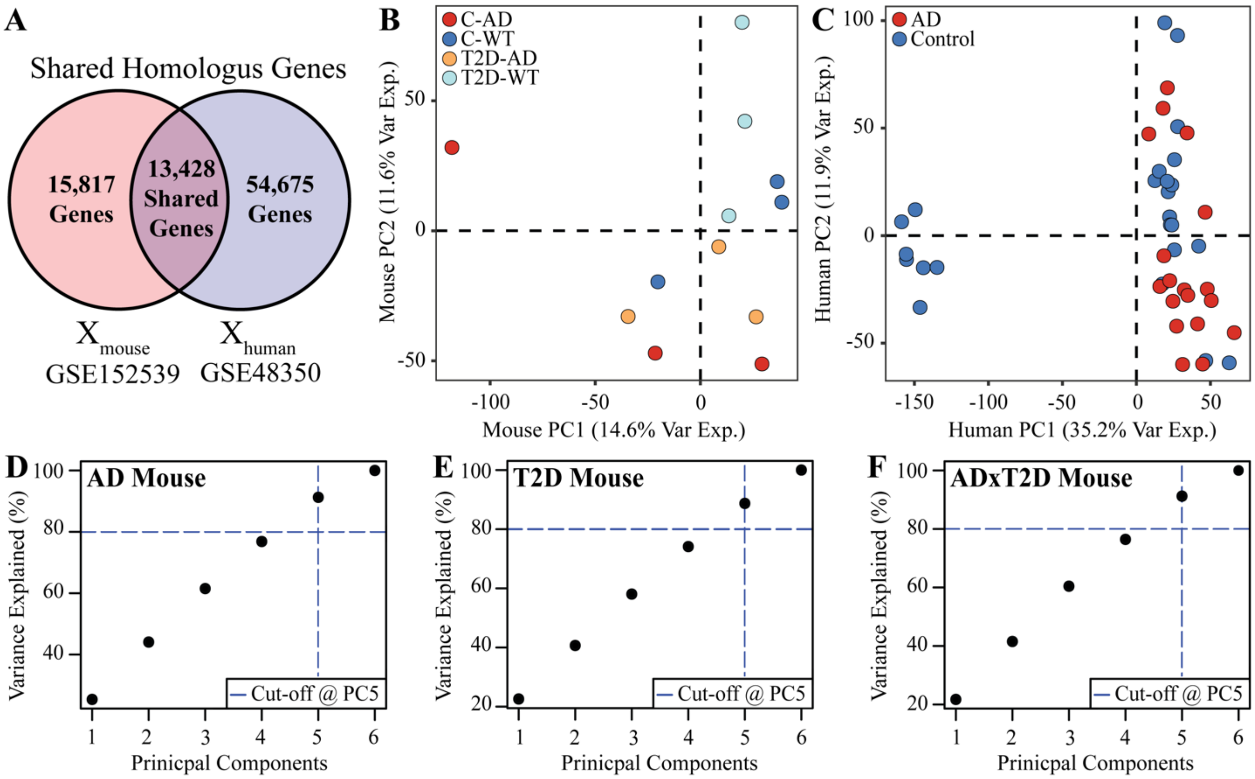

Supplementary Figure S1. Overview of the publicly available human and mouse data. (A) The mouse and human dataset share 13,428 homologous gene pairs. **(B)** PCA of the four mouse conditions. **(C)** PCA of the human and control groups. **(D-F)** Cumulative variance explained plots for each of the respective mouse conditions, each with a PC cut-off of 5 PCs.

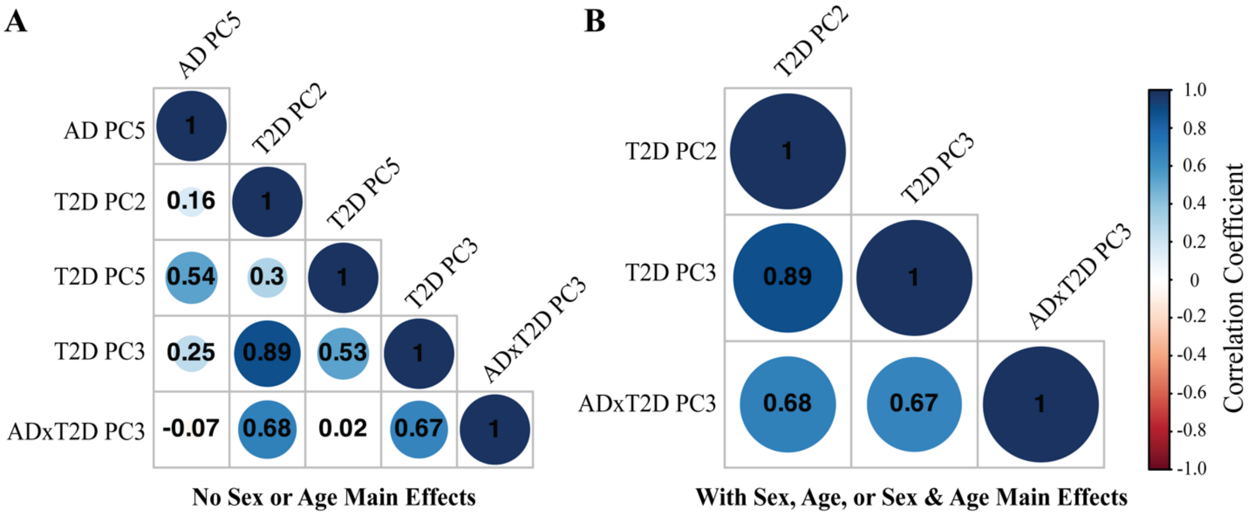

Supplementary Figure S2. Spearman correlation plots of the selected PCs from LASSO. (A) Correlation plot for the model without sex or age as included main effects. **(B)** Correlation plot for models with sex, age, or sex and age as included main effects in the LASSO model. Models with sex, age, or sex and age main effects all contained the same selected PCs from LASSO.

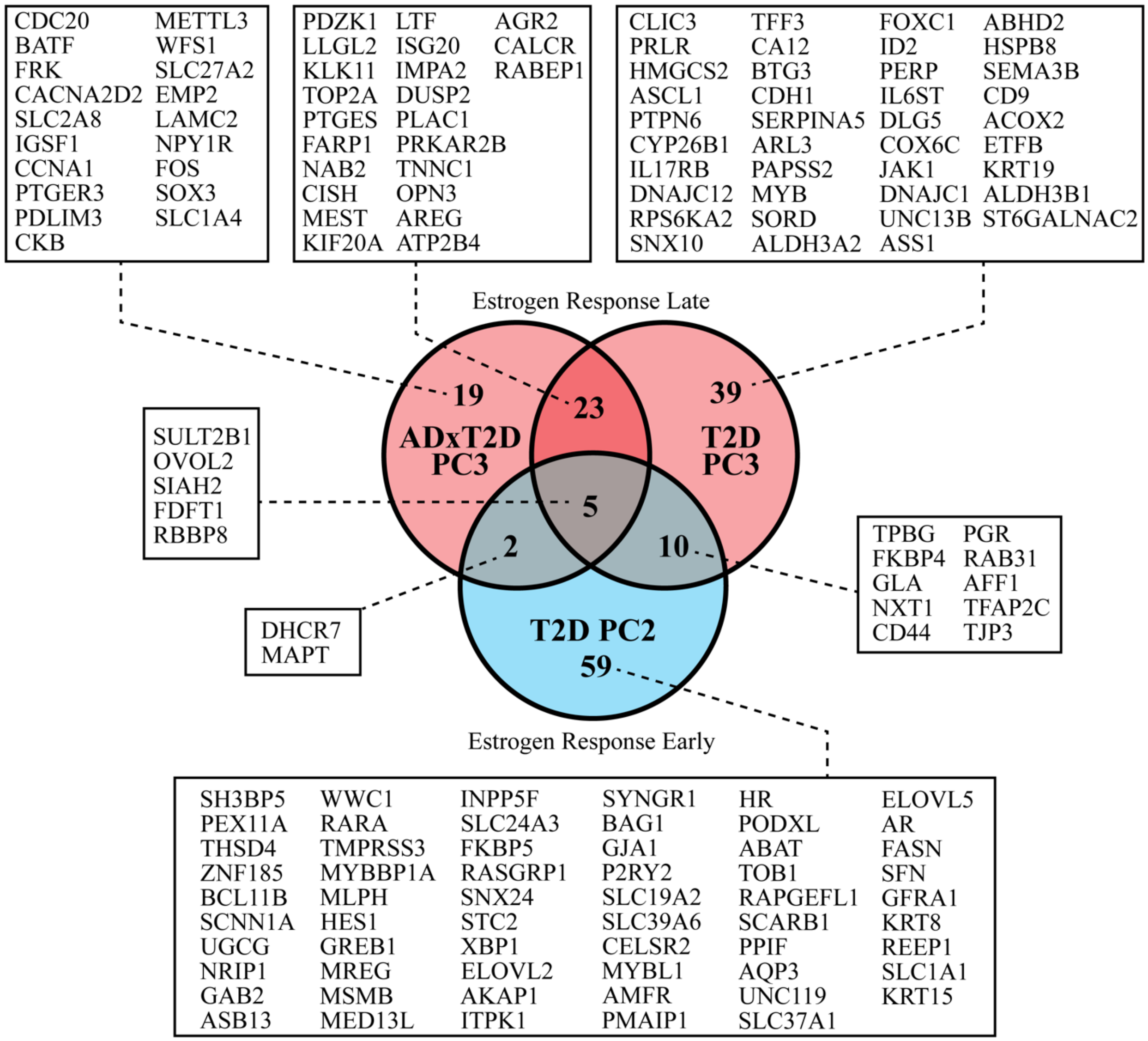

Supplementary Figure S3. Number of shared genes enriched for estrogen pathways. Mouse T2D PC3 and ADxT2D PC3 identified "Estrogen Response Late" to be enriched in AD (red), whereas mouse T2D PC2 was enriched for "Estrogen Response Early" in human control groups (blue). Overlapped genes represent gene signatures that were enriched in more than one mouse PC.
